# Accuracy of Gallium-68 Pentixafor Positron Emission Tomography–Computed Tomography for Subtyping Diagnosis of Primary Aldosteronism

**DOI:** 10.1001/jamanetworkopen.2022.55609

**Published:** 2023-02-16

**Authors:** Jinbo Hu, Tingting Xu, Hang Shen, Ying Song, Jun Yang, Aipin Zhang, Haoyuan Ding, Naiguo Xing, Zhuoyuan Li, Lin Qiu, Linqiang Ma, Yi Yang, Zhengping Feng, Zhipeng Du, Wenwen He, Yue Sun, Jun Cai, Qifu Li, Yue Chen, Shumin Yang

**Affiliations:** 1 Department of Endocrinology, The First Affiliated Hospital of Chongqing Medical University, Chongqing, China; 2 Department of Nuclear Medicine, The Affiliated Hospital of Southwest Medical University, Luzhou, Sichuan, China; 3 Nuclear Medicine and Molecular Imaging Key Laboratory of Sichuan Province, Luzhou, Sichuan, China; 4 Institute of Nuclear Medicine, Southwest Medical University, Luzhou, Sichuan, China; 5 Department of Medicine, Monash University, Clayton, Victoria, Australia; 6 Centre for Endocrinology and Metabolism, Hudson Institute of Medical Research, Clayton, Victoria, Australia; 7 Graduate Administration Office, The First Affiliated Hospital of Chongqing Medical University, Chongqing, China; 8 Hypertension Center, Fuwai Hospital, State Key Laboratory of Cardiovascular Disease, National Center for Cardiovascular Diseases, Chinese Academy of Medical Sciences and Peking Union Medical College, Beijing, China

## Abstract

**Importance:**

Adrenal vein sampling (AVS) is the recommended procedure for subtyping primary aldosteronism (PA) as unilateral PA (UPA) or bilateral PA (BPA), with different treatment needed for each: adrenalectomy for UPA and medication for BPA. However, AVS is invasive and technically difficult, and how to subtype PA noninvasively is currently a great challenge.

**Objective:**

To evaluate the accuracy of gallium-68 pentixafor positron emission tomography–computed tomography (PET-CT) in subtyping PA using AVS as a reference standard.

**Design, Setting, and Participants:**

This diagnostic study was conducted at a tertiary hospital in China among patients diagnosed with PA. Enrollment was started in November 2021, with follow-up ending in May 2022.

**Exposures:**

**:** Patients were recruited to undergo gallium-68 pentixafor PET-CT and AVS.

**Main Outcomes and Measures:**

Maximum standardized uptake value (SUVmax) of each adrenal gland during PET-CT was measured to calculate the lateralization index of SUVmax. Area under the receiver operating characteristic curve (AUROC), specificity, and sensitivity were used to analyze the accuracy of the lateralization index based on SUVmax for subtyping PA.

**Results:**

Among 100 patients with PA who completed the study (47 female [47.0%] and 53 male [53.0%]; median [IQR] age, 49 [38-56] years), 43 individuals had UPA and 57 individuals had BPA. Aldosterone-cortisol ratio (Spearman ρ = 0.26; *P* < .001) in adrenal veins was positively correlated with SUVmax of adrenal glands at 10 minutes during PET-CT. Using lateralization index based on SUVmax at 10 minutes to identify UPA, the AUROC was 0.90 (95% CI, 0.83-0.97). A cutoff value for lateralization index based on SUVmax at 10 minutes set at 1.65 conferred a specificity of 1.00 (95% CI, 0.94-1.00) and sensitivity of 0.77 (95% CI, 0.61-0.88). The diagnostic concordance rate of PET-CT and AVS was 90 patients (90.0%) compared with 54 patients (54.0%) between traditional CT and AVS.

**Conclusions and Relevance:**

This study found good diagnostic accuracy of gallium-68 pentixafor PET-CT in differentiating UPA from BPA. These findings suggest that gallium-68 pentixafor PET-CT may be used to avoid invasive AVS in some patients with PA.

## Key Points

**Question:**

What is the accuracy of gallium-68 pentixafor positron emission tomography–computed tomography (PET-CT) in subtyping diagnosis of primary aldosteronism (PA)?

**Findings:**

In this diagnostic study of 100 patients with PA using adrenal vein sampling as a reference standard, the area under the receiver operating characteristic curve of PET-CT was 0.90, conferring a specificity of 1.00 and sensitivity of 0.77.

**Meaning:**

These findings suggest that gallium-68 pentixafor PET-CT may be a promising noninvasive subtyping method for patients with PA.

## Teaser

This diagnostic study investigates the accuracy of gallium-68 pentixafor positron emission tomography–computed tomography vs adrenal vein sampling in subtyping diagnosis of primary aldosteronism.

## Introduction

Primary aldosteronism (PA) is one of the most common causes of secondary hypertension, accounting for 5% to 22% of incidents of hypertension.^1,2,3,4^ Autonomous secretion of aldosterone in PA is caused by cortical lesions on 1 (unilateral PA [UPA]) or both adrenal glands (bilateral PA [BPA]). For patients with confirmed PA, the differentiation of UPA from BPA is essential because surgical treatment is recommended for UPA while an oral mineralocorticoid receptor antagonist, such as spironolactone, is the first line of treatment for BPA.

Currently, the main subtyping methods include adrenal computed tomography (CT) and adrenal vein sampling (AVS). Because the accuracy of CT is 50% to 70%,^5,6,7^ guidelines suggest that most patients with PA who are willing to have surgery should undergo AVS, which is widely accepted as the criterion standard to confirm the subtyping classification.^8,9^ However, AVS is an invasive test that is technically challenging, labor intensive, and expensive, which limits its clinical use.^10^ Finding strategies to subtype PA by noninvasive means is currently a clinical challenge. In this context, functional imaging techniques are of great interest.

^11^C-metomidate positron emission tomography (PET) targeting aldosterone synthase has been investigated, but the reported results have not been consistent.^11,12,13,14^ Furthermore, application of this method is problematic due to the need for several days of pretreatment with dexamethasone.^15^ A 2018 study^16^ found that adrenal CXC chemokine receptor type 4 (CXCR4) expression was significantly higher in aldosterone-producing adenomas (APAs) than normal adrenal tissue or nonfunctional tumors. Findings from case series studies^17,18^ of patients with APA suggest that Pentixafor, a specific ligand for CXCR4, labeled with ^68^Ga may provide clinical utility in PA subtype classification. In this study, we prospectively recruited patients with confirmed PA and evaluated the diagnostic accuracy of gallium-68 pentixafor PET-CT in differentiating UPA from BPA using AVS as the reference standard.

## Methods

This was a prospective diagnostic accuracy study designed and reported following the Standards for Reporting of Diagnostic Accuracy (STARD) reporting guideline. The Ethics Committee of the First Affiliated Hospital of Chongqing Medical University approved the protocol. Informed written consent was obtained from each participant.

### Study Design and Participants

This study was conducted at the First Affiliated Hospital of Chongqing Medical University in China from Nov 2021 to May 2022. Patients diagnosed with PA were recruited. Inclusion and exclusion criteria are provided in the eAppendix in Supplement 1.

### Diagnosis of PA

All patients underwent PA screening by plasma aldosterone–renin ratio (ARR). The screening test was considered positive when the ARR was 3.16 ng · dL^−1^/pg · mL^−1^ (2.0 ng · dL^−1^/μIU · mL^−1^).^19,20^ Detailed screening methods are provided in the eAppendix in Supplement 1.

Patients who tested positive proceeded to confirmatory testing with a captopril challenge test and seated saline infusion test. Aldosterone fluctuations can be associated with false-negative ARR screening,^21,22^ so for patients who tested negative with the ARR, if PA was strongly suspected based on young age, hypokalemia, resistant hypertension, or typical adrenal adenomas on CT scan, they also proceeded to the confirmatory test. PA was confirmed if they had a positive result in the confirmatory test (criteria provided in the eAppendix in Supplement 1). In patients with an onset of confirmed PA earlier than age 20 years or in those with a family history of PA, genetic testing for familial hyperaldosteronism was performed.

### ^68^Ga-Pentixafor PET-CT Scanning and Image Analysis

Patients confirmed with PA underwent PET-CT examination. Detailed methods are provided in the eAppendix in Supplement 1. In brief, local PET-CT scanning of the upper abdomen was performed at 10 and 40 minutes after injection of the tracer. Noncontrast CT images were acquired over the upper abdomen. Adrenal lesion on CT included nodule (defined as round or oval, with smooth margins, well defined, and ≥4 mm in diameter) and hyperplasia (if adrenal gland thickness measured ≥10 mm in diameter).^23,24,25^ Lesions shown on CT or those with no abnormality on CT but suspected of increased tracer uptake on PET were located as regions of interest, and maximal standardized uptake value (SUVmax) was measured in these regions. For adrenal glands with neither morphological changes nor increased tracer uptake, SUVmax within each adrenal gland was also recorded. A mean SUVmax of 5 round spheres with a diameter of 2 cm was selected from the liver as the whole-body background.

Lateralization index (LI) based on SUVmax at 10 minutes and 40 minutes, dominant side of SUVmax at 10 minutes and 40 minutes, and dominant side of SUVmax adjusted by liver at 10 minutes and 40 minutes were calculated for the diagnostic accuracy analysis. The side with higher SUVmax in both adrenal glands is the dominant side. LI based on SUVmax was defined as (SUVmax of dominant side)/(SUVmax of nondominant side). SUVmax adjusted by liver was defined as (SUVmax of adrenal)/(SUVmax of liver). Dominant side of SUVmax adjusted by liver was defined as (SUVmax of dominant side in adrenal)/(SUVmax of liver).

### AVS

Patients underwent AVS within 3 months of completing gallium-68 pentixafor PET-CT to determine the lateralization of aldosterone hypersecretion. AVS without adrenocorticotropic hormone stimulation was performed in the morning between 8:00 am and 12:00 pm. In brief, successful cannulation of the adrenal veins was defined as a selectivity index of 2 or greater. A diagnosis of UPA was made if LI based on AVS was 4 or greater or 2 to 4 in combination with contralateral suppression or CT showing a typical adenoma on the dominant side, while those with LI based on AVS of less than 2 or 2 to 4 without meeting the previously described criteria were diagnosed as BPA.^26,27^ Detailed methods and criteria are provided in the eAppendix in Supplement 1.

### Follow-ups

At least 1 follow-up was done in the 1 to 6 months after surgery. Biochemical remission was defined based on the Primary Aldosteronism Surgery Outcome criteria.^28^

### Measurements and Assay Methods

Blood pressure was measured according to the European Society of Hypertension and European Society of Cardiology guidelines for the management of arterial hypertension.^29^ Plasma renin concentration and plasma aldosterone concentration (PAC) were measured with an automated chemiluminescence immunoassay (Liason; DiaSorin). Details are provided in the eAppendix in Supplement 1.

### Statistical Analysis

Sample size calculation and detailed statistical methods are provided in the eAppendix in Supplement 1. Area under the receiver operating characteristic curve (AUROC), sensitivity, specificity, positive predictive value, negative predictive value, and Youden index were calculated to evaluate accuracy. Missing data were imputed with the multivariate imputation by chained equations algorithm.^30^ Regression models were used to estimate missing values. The extent of missing data of study variables is provided in eTable 1 in Supplement 1. *P* values were 2-sided, and *P* values < .05 were considered statistically significant. PASS statistical software version 11.0.7 (NCSS) was used to calculate sample size. Imputation of missing data was conducted using R statistical software version 4.0.1 (R Project for Statistical Computing). Statistical analyses were performed with SPSS statistical software version 23.0 (IBM).

## Results

### Clinical Characteristics of the Patients

**Figure 1.**
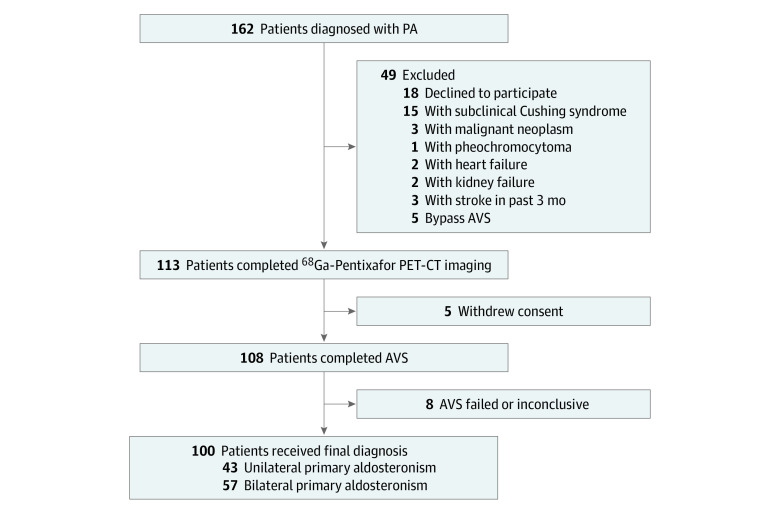
Study Flowchart AVS indicates adrenal vein sampling; PA, primary aldosteronism; PET-CT, positron emission tomography–computed tomography.

During the study, 162 patients diagnosed with PA were screened and 62 patients were excluded. Finally, 100 patients (47 female [47.0%] and 53 male [53.0%]; median [IQR] age, 49 [38-56] years), completed the study, including 43 individuals with UPA and 57 individuals with BPA (Figure 1). Compared with patients in the BPA group, patients with UPA had higher diastolic blood pressure, PAC, ARR, and PAC postconfirmatory test results and lower body mass index (calculated as weight in kilograms divided by height in meters squared) and plasma renin concentration (Table 1).

**Table 1.** Clinical Characteristics of Included Patients

Characteristic	Patients, Median (IQR)			*P* value
	Total (N = 100)	UPA (n = 43)	BPA (n = 57)	
Age, y	49 (38-56)	47 (37-55)	51 (40-57)	.20
Sex, No. (%)				.93
Male	53 (53)	23 (53.5)	30 (52.6)	
Female	47 (47)	20 (46.5)	27 (47.4)	
BMI, mean (SD)	25.4 (3.3)	24.3 (3.0)	25.9 (3.4)	.01
SBP, mean (SD), mm Hg	152 (23)	156 (19)	150 (25)	.16
DBP, mm Hg	92 (85-104)	96 (87-110)	90 (83-102)	.04
Serum potassium+, mean (SD), mEq/L^a^	3.3 (0.7)	3.2 (0.8)	3.5 (0.6)	.06
PAC, ng/dL	21.2 (15.4-31.1)	24.2 (15.4-37.1)	19.7 (15.4-23.4)	.03
PRC, pg/mL	2.3 (0.7-4.3)	1.11 (0.39-3.25)	3.0 (1.0-5.0)	<.001
ARR, ng · dl^−1^/pg · mL^−1^	10.0 (4.9-30.4)	24.2 (8.4-60.0)	7.3 (4.2-17.2)	<.001
PAC after CCT, ng/dL	17.0 (12.5-28.2)	27.1 (16.7-33.6)	14.1 (11.2-18.7)	<.001
PAC after SSIT, ng/dL	11.4 (9.1-19.2)	17.1 (11.3-31.2)	10.3 (8.4-12.8)	<.001
CT scan findings, No. (%)				.04
Bilateral normal	9 (9.0)	1 (2.3)	8 (14.0)	
Bilateral lesion	17 (17.0)	5 (11.6)	12 (21.1)	
Unilateral lesion	74 (74.0)	37 (86.1)	37 (64.9)	

Abbreviations: BMI, body mass index (calculated as weight in kilograms divided by height in meters squared); BPA, bilateral primary aldosteronism; CCT, captopril challenge test; CT, computed tomography; DBP, diastolic blood pressure; PAC, plasma aldosterone concentration; PRC, plasma renin concentration; SBP, systolic blood pressure; SSIT, seated saline infusion test; UPA, unilateral primary aldosteronism.

SI conversions: To convert aldosterone to picomoles per liter, multiply by 27.74; potassium to millimoles per liter, multiply by 1.0; renin to picomoles per liter, multiply by 0.0237.

^a^ Serum potassium was the lowest level in the medical history before treatment of hypokalemia.

### Results of AVS and SUVmax and Their Correlation

Patients with UPA compared with patients in the BPA group had higher median (IQR) adrenal SUVmax (eg, at 10 minutes: 12.90 [9.90-17.50] vs 7.30 [5.90-9.55]), adrenal vein PAC (1636.67 [678.00-3515.00] ng/dL vs 595.60 [271.15-1637.50] ng/dL) and aldosterone-cortisol ratio (9.77 [3.93-17.82] vs 3.52 [1.82-6.92]) on the dominant side and lower adrenal SUVmax (eg, at 10 minutes: 4.90 [3.90-7.30] vs 6.20 [5.10-7.30]), adrenal vein PAC (169.13 [55.70-290.00] ng/dL vs 648.67 [196.00-1142.75] ng/dL), and aldosterone-cortisol ratio on the nondominant side (0.65 [0.28-1.41] vs 1.93 [1.14-3.45]). When the adrenal SUVmax was adjusted by liver, the UPA group had a higher adjusted SUVmax on the dominant side but not a lower adjusted SUVmax on the nondominant side. In the UPA group, median (IQR) LI based on AVS was more than 7 times higher than in the BPA group (13.17 [4.42-33.52] vs 1.82 [1.29-2.48]), and median (IQR) LI based on SUVmax at 10 minutes was 2 times higher than that in the BPA group (2.36 [1.67-3.25] vs 1.19 [1.06-1.35]) (Table 2).

**Table 2.** Parameters of Gallium-68 Pentixafor Positron Emission Tomography–Computed Tomography and Adrenal Vein Sampling

Parameter	Median (IQR)			*P* value
	Total (N = 100)	UPA (n = 43)	BPA (n = 57)	
Dominant side				
Of SUVmax at 10 min	9.25 (6.43-12.40)	12.90 (9.90-17.50)	7.30 (5.90-9.55)	<.001
Of 10 min SUVmax adjusted by liver	4.05 (2.92-6.65)	7.0 (4.77-8.69)	3.40 (2.46-4.15)	<.001
Of SUVmax at 40 min	6.25 (4.70-11.05)	11.20 (7.50-17.30)	5.40 (3.95-6.45)	<.001
Of 40 min SUVmax adjusted by liver	4.48 (2.86-8.31)	8.57 (5.60-10.83)	3.40 (2.36-4.56)	<.001
Nondominant side				
Of SUVmax at 10 min	5.60 (4.50-7.30)	4.90 (3.90-7.30)	6.20 (5.10-7.30)	.03
Of 10 min SUVmax adjusted by liver	2.63 (2.05-3.26)	2.61 (2.05-3.23)	2.78 (2.04-3.27)	.79
Of SUVmax at 40 min	3.95 (3.10-5.18)	3.60 (2.90-5.0)	4.50 (3.50-5.25)	.05
Of 40 min SUVmax adjusted by liver	2.86 (2.10-3.58)	2.71 (2.18-3.38)	2.93 (2.05-3.65)	.51
LI based on SUVmax				
At 10 min	1.35 (1.16-2.20)	2.36 (1.67-3.25)	1.19 (1.06-1.35)	<.001
At 40 min	1.36 (1.15-2.63)	2.89 (1.87-4.55)	1.20 (1.10-1.31)	<.001
Dominant side				
Of PAC, ng/dL	1081.50 (404.25-2402.92)	1636.67 (678.00-3515.00)	595.60 (271.15-1637.50)	.001
Of PCC, μg/dL	184.98 (53.75-524.63)	188.48 (73.27-413.33)	181.49 (45.53-557.92)	.69
Of ACR	4.48 (2.52-12.21)	9.77 (3.93-17.82)	3.52 (1.82-6.92)	<.001
Non-dominant side				
Of PAC, ng/dL	290.75 (98.79-760.13)	169.13 (55.70-290.00)	648.67 (196.00-1142.75)	<.001
Of PCC, μg/dL	265.20 (107.82-589.28)	252.19 (127.50-527.97)	278.53 (73.46-602.78)	.97
Of ACR	1.35 (0.57-2.73)	0.65 (0.28-1.41)	1.93 (1.14-3.45)	<.001
LI based on AVS	2.67 (1.72-9.46)	13.17 (4.42-33.52)	1.82 (1.29-2.48)	<.001

Abbreviations: ACR, aldosterone-to-cortisol ratio; AVS, adrenal vein sampling; BPA, bilateral primary aldosteronism; LI, lateralization index; PAC, plasma aldosterone concentration; PCC, plasma cortisol concentration; SUVmax, maximum standardized uptake value; UPA, unilateral primary aldosteronism.

SI conversions: To convert aldosterone to picomoles per liter, multiply by 27.74; cortisol to nanomoles per liter, multiply by 27.588.

PAC (Spearman ρ = 0.13; *P* = .049) and aldosterone-cortisol ratio (Spearman ρ = 0.26; *P* < .001) in adrenal veins were positively correlated with SUVmax of adrenal glands at 10 minutes during the PET-CT. Cortisol concentration in adrenal veins was not correlated with SUVmax of adrenal glands at 10 minutes (Spearman ρ = −0.10; *P* = .31) or 40 minutes (Spearman ρ = −0.13; *P* = .21). LI based on AVS was positively correlated with LI based on SUVmax at 10 minutes (Spearman ρ = .56; *P* <.001) and SUVmax at 40 minutes (Spearman ρ = 0.52; *P* < .001) (eFigure 1 in Supplement 1).

### Diagnostic Accuracy of Gallium-68 Pentixafor PET-CT

**Figure 2.**
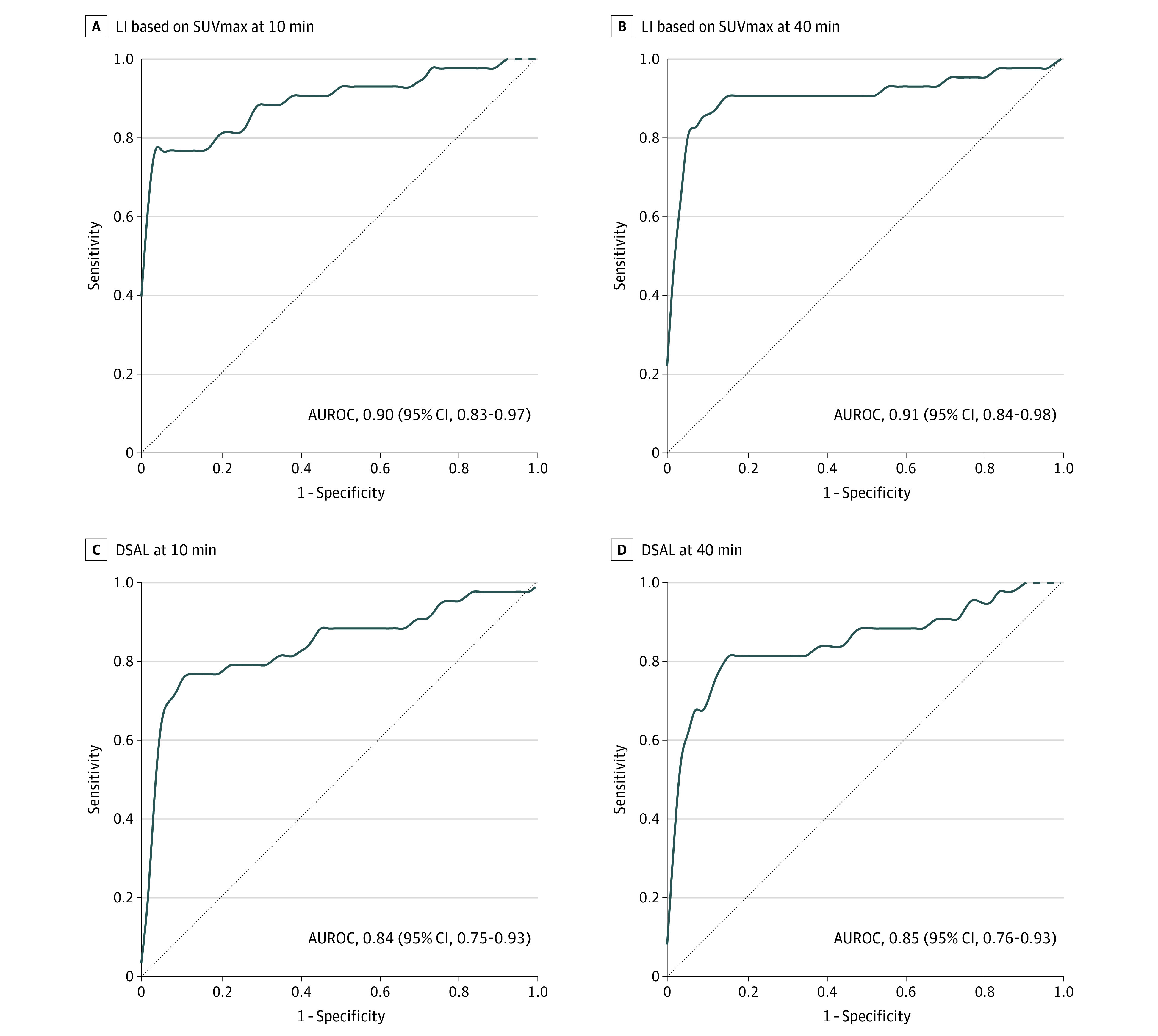
Receiver Operating Characteristic Curves for Diagnosis of Unilateral Primary Aldosteronism AUROC indicates area under the receiver operating characteristic curve; DSAL, dominant side of maximum standardized uptake value adjusted by liver; LI, lateralization index; SUVmax, maximum standardized uptake value.

To diagnose UPA, LI based on SUVmax (Figure 2) had a higher AUROC (eg, at 10 minutes: 0.90 [95% CI, 0.83-0.97]) than that of the dominant side of SUVmax (eg, 10 minutes: 0.82 [95% CI, 0.73-0.91]) (eFigure 2 in Supplement 1) and liver-adjusted SUVmax (eg, at 10 minutes: 0.84 [95% CI, 0.75-0.93]) (Figure 2). To achieve the maximized Youden index, the optimal cutoff of LI based on SUVmax at 10 minutes was 1.65, with a sensitivity of 0.77 (95% CI, 0.61-0.88) and a specificity of 1.00 (95% CI, 0.94-1.00). Using this cutoff, 33 patients with UPA (76.7%) would be accurately subtyped, while 10 patients (23.3%) would be missed; however, no patients with BPA would be misdiagnosed as UPA. With a lower cutoff of 1.10, the sensitivity was increased to 0.98 (95% CI, 0.88-1.00) with a decreased specificity of 0.26 (95% CI, 0.16-0.40) (Table 3). Representative images of gallium-68 pentixafor PET-CT imaging in patients with PA are shown in eFigure 3 in Supplement 1.

**Table 3.** Diagnostic Accuracy for Primary Aldosteronism Subtyping Using LI Based on SUVmax

Cutoff	No.				Sensitivity (95% CI)	Specificity (95% CI)	YI	PPV (95% CI)	NPV (95% CI)
	TP	FP	FN	TN					
LI based on SUVmax at 10 min									
1.10	42	42	1	15	0.98 (0.88-1.00)	0.26 (0.16-0.40)	0.24	0.50 (0.39-0.61)	0.94 (0.70-1.00)
1.56	33	4	10	53	0.77 (0.61-0.88)	0.93 (0.83-0.98)	0.7	0.89 (0.75-0.979)	0.84 (0.73-0.92)
1.65	33	0	10	57	0.77 (0.61-0.88)	1.00 (0.94-1.00)	0.77	1.00 (0.89-1.00)	0.85 (0.74-0.93)
LI based on SUVmax at 40 min									
1.12	40	40	3	17	0.93 (0.81-0.99)	0.30 (0.18-0.43)	0.23	0.50 (0.39-0.61)	0.85 (0.62-0.97)
1.57	37	5	6	52	0.86 (0.72-0.95)	0.91 (0.81-0.97)	0.77	0.88 (0.74-0.96)	0.90 (0.79-0.96)
3.15	19	0	24	57	0.44 (0.29-0.60)	1.00 (0.94-1.00)	0.44	1.00 (0.82-1.00)	0.70 (0.59-0.80)

Abbreviations: FN, false negative; FP, false positive; LI, lateralization index; NPV, negative predictive value; PPV, positive predictive value; SUVmax, maximum standardized uptake value; TN, true negative; TP, true positive; YI, Youden index.

To achieve the maximized Youden index, the cutoff of LI based on SUVmax at 40 minutes was 1.57, with a sensitivity of 0.86 (95% CI, 0.72-0.95) and a specificity of 0.91 (95% CI, 0.81-0.97). Using this cutoff, 5 patients with BPA (8.8%) would be misdiagnosed as UPA. Given that patients with UPA receive surgical treatment, the diagnostic specificity should be as high as possible to avoid unnecessary adrenalectomy. To increase the specificity to 1.00, a cutoff of 3.15 was used, which had a lower sensitivity than the 10-minute SUVmax LI cutoff of 1.65 (0.44 [95% CI, 0.29-0.60]) (Table 3). Cutoff values of SUVmax and liver-adjusted SUVmax showed inferior accuracy compared with LI based on SUVmax (eTable 2 in Supplement 1).

Given that the final subtyping diagnosis must be specific to the left or right adrenal gland and not just distinguish unilateral from bilateral, we further analyzed the concordance rate of AVS and PET-CT diagnosis (eTable 3 in Supplement 1). All patients with UPA identified by PET-CT (using LI based on SUVmax at 10 minutes ≥1.65 for diagnosis) were confirmed as having ipsilateral UPA by AVS. No patient had AVS-determined lateralization that was contralateral to the lesion on PET-CT, and 57 of 67 patients with BPA identified by PET-CT were confirmed as having BPA by AVS. Therefore, the total concordance rate of AVS and PET-CT reached 90 patients (90.0%). The concordance rate increased to 40 of 40 patients (100%) with unilateral adrenal nodules 10 mm or greater in diameter on CT (eTable 4 in Supplement 1), which was higher than that between traditional adrenal CT and AVS (55 patients [55.0%]) (eTable 5 in Supplement 1). Using LI based on SUVmax at 40 minutes for subtyping diagnosis, the total concordance rate was similar to that of LI based on SUVmax at 10 minutes (89 patients [89.0%]); however, 5 patients with BPA would be misdiagnosed as UPA and undergo unnecessary surgery (eTable 6 in Supplement 1).

The 10 patients with UPA missed by PET-CT had unilateral hyperplasia, a unilateral nodule less than 10 mm, bilateral lesions, or normal-appearing adrenal glands on CT (eTables 7 and 8 in Supplement 1). PET-CT was more likely to accurately subtype UPA in patients who had a typical unilateral nodule (≥10 mm) on CT.

### Surgical Outcomes

Among 31 patients with UPA diagnosed by AVS who underwent laparoscopic adrenalectomy, pathological diagnosis confirmed 26 patients with APA (19 patients with a nodule ≥10 mm and 7 patients with a nodule <10mm), 4 patients with nodular hyperplasia (2 patients with a nodule ≥10 mm and 2 patients with a nodule <10 mm), and 1 patient with diffuse hyperplasia. In total, 21 of 21 patients (100%) with a large nodule (≥10 mm), 5 of 9 patients (55.6%) with a micronodule (<10 mm), and 1 patient with diffuse hyperplasia showed positive imaging on PET-CT. The smallest aldosterone-producing nodule detected on PET-CT was 8 mm in diameter. Nearly all patients with UPA diagnosed by AVS who underwent laparoscopic adrenalectomy (30 patients [96.7%]) had complete biochemical remission after surgery. There was 1 patient who had a concordant diagnosis of left-sided APA by AVS and PET-CT and was confirmed with nodular hyperplasia by pathological examination who showed partial biochemical remission (eTable 9 in Supplement 1).

## Discussion

This diagnostic study found a good diagnostic accuracy of gallium-68 pentixafor PET-CT in differentiating UPA from BPA. The gallium-68 pentixafor PET-CT was concordant with AVS outcomes in 90% of patients with PA, and the concordance rate increased to 100% among patients with unilateral adrenal nodules greater than 10 mm in diameter on CT. These findings suggest that gallium-68 pentixafor PET-CT may be a useful subtyping diagnostic method in some patients with PA.

The poor concordance of AVS and adrenal CT found in our study concurred with findings from previous reports.^5,7,31,32^ A number of scores based on clinical features have been proposed to spare AVS in patients with a high probability of having UPA or BPA,^5,33,34,35,36,37,38,39,40,41,42^ but none of these scores showed an accuracy high enough to justify their clinical use. Functional imaging techniques, such as iodocholesterol scintigraphy and ^11^C-metomidate PET-CT, have been investigated in the last decade, but reported sensitivity and specificity were not sufficient to replace AVS.^11,12,13,14,43^ Furthermore, the requirement for an on-site cyclotron and need for pretreatment with dexamethasone days before examination further limited their use. On the contrary, no special preparation is required for gallium-68 pentixafor imaging, and our data revealed a good diagnostic accuracy using this method.

There were 2 previous studies^16,17^ that reported on the use of gallium-68 pentixafor PET-CT imaging in patients with PA. In Heinze et al,^16^ using gallium-68 pentixafor uptake in normal adrenal glands as reference values, researchers investigated gallium-68 pentixafor imaging in 9 patients with APA. Using SUVmax to identify APA, the AUROC was reported to be 0.964, and a cutoff value for SUVmax of 4.9 revealed a sensitivity of 88.9% and a specificity of 87.2% for diagnosing APA.^16^ In Ding et al,^17^ gallium-68 pentixafor imaging was performed in 25 patients with APA, 4 patients with BPA, and 10 patients with nonfunctional adrenal adenoma.^17^ Data revealed that a cutoff value for LI based on SUVmax (25-30 minutes after injection of the tracer) of 2.12 yielded a sensitivity of 100% and a specificity of 92.9%, whereas a cutoff value for the dominant side of SUVmax adjusted by liver of 2.36 reached 100% for sensitivity and specificity. In our data, the dominant side of SUVmax (and liver-adjusted SUVmax) in the UPA group was higher than in the BPA group, but LI based on SUVmax at 10 minutes had better accuracy than SUVmax and liver-adjusted SUVmax. Heinze et al^16^ found that the expression of CXCR4 was increased in patients with APA compared with those with normal adrenal tissue and those with nonfunctioning adenoma, but whether the expression of CXCR4 was increased in hyperplastic adrenal tissue in patients with PA was unknown. If the expression of CXCR4 is also increased in hyperplastic tissue, then hypothetically, the SUVmax ratio of 2 adrenal glands (namely, LI of SUVmax in our study) should be superior to SUVmax, and our data supported this hypothesis.

In our study, the SUVmax was recorded in early and delayed PET imaging (10 minutes and 40 minutes after injection of the tracer, respectively). The optimal cutoff of LI based on SUVmax at 10 minutes was 1.65, and no patients with BPA would be misdiagnosed as having UPA at this cutoff. However, if using SUVmax at 40 minutes for diagnosis with the optimal cutoff, 9% of patients with BPA would have undergone an unnecessary surgery. To increase the specificity to 1.00, the LI cutoff based on SUVmax at 40 minutes needed to be increased to 3.15, which was associated with a lower sensitivity than the 10-minute SUVmax LI. Our results suggest that SUVmax of early PET imaging may be better than SUVmax of delayed imaging.

In our study, the concordance rate of PET-CT and AVS was 100% in patients with a unilateral adrenal nodule 10 mm or greater on CT, while in patients without a typical unilateral nodule, the sensitivity of PET imaging was decreased, suggesting the limited spatial resolution of this approach. Previous studies reported high expression of CXCR4 in 71% to 96% of patients with APA.^16,17^ van de Wiel et al^44^ performed immunohistochemistry analysis on adrenal nodules from patients with UPA and reported that every *CYP11B2*-positive nodule showed CXCR4 staining. Notably, almost half of these nodules were less than 1 cm, suggesting that gallium-68 pentixafor PET-CT imaging may facilitate the identification of some hyperfunctioning micronodules. However, nearly half of patients with UPA with micronodules were missed by PET-CT in our study, suggesting that the sensitivity of this method may not be ideal in patients with small lesions.

The strengths of our study included a prospective design and the use of a rigorous protocol for the diagnosis and subtyping of PA. In addition, gallium-68 pentixafor PET-CT was evaluated by 2 independent nuclear medicine physicians (T.X. and H.D.) who were blinded to AVS results. Furthermore, more than half of patients enrolled in the study had BPA, which is close to the subtype distribution found clinically and supports generalized applicability of the method in patients with UPA and BPA.

### Limitations

This study has several limitations. Our data showed a high specificity of 100% with the optimal cutoff, but this was tested in only 100 patients with PA. Although we prospectively set a sample size, our findings need to be further verified in a larger population. Another limitation was that some patients with UPA in our study did not undergo surgery. Although AVS is currently recognized as the criterion standard for subtyping of PA, surgery and follow-up would further confirm the diagnosis of UPA. Results may also be different for other AVS procedures, such simultaneous AVS or AVS with adrenocorticotropic hormone stimulation. In addition, given that increased CXCR4 expression was found in cortisol-producing adenomas^16^ and patients with Cushing syndrome were reported to show a positive gallium-68 pentixafor PET-CT result in a previous study,^45^ patients with PA concurrent with Cushing syndrome were excluded in our study. Therefore, the accuracy of gallium-68 pentixafor PET-CT in patients with PA concurrent with autonomous cortisol secretion needs further study.

## Conclusions

This diagnostic study’s findings suggest that using gallium-68 pentixafor PET-CT may facilitate PA subtyping diagnosis, especially in patients with a typical unilateral adrenal nodule greater than 10 mm. Although this approach may not fully replace AVS, it may be associated with a substantially reduced number of AVS procedures required for selected patients. Considering the universal need for safe, accurate, and noninvasive alternatives to AVS, gallium-68 pentixafor PET-CT may represent a novel and promising tool for PA subtyping, although it warrants further evaluation in a larger population.

## Notes

Supplement 1. **eAppendix.** Supplementary Methods **eFigure 1.** Scatter Plots Showing Correlation of Maximum Standardized Uptake Value With Adrenal Vein Sampling Parameters **eFigure 2.** Receiver Operating Characteristic Curve of Dominant Side of Maximum Standardized Uptake Value During Gallium-68 Pentixafor PET-CT for Diagnosis of Unilateral Primary Aldosteronism **eFigure 3.** Representative Images of Gallium-68 Pentixafor PET-CT Imaging in Patients With Primary Aldosteronism **eTable 1.** Proportion of Missing Data in Study Variables **eTable 2.** Diagnostic Accuracy of Adjusted Maximum Standardized Uptake Value at Different Cutoff Values **eTable 3.** Concordance of Gallium-68 Pentixafor PET-CT and Adrenal Vein Sampling in All Patients With PA **eTable 4.** Concordance of Gallium-68 Pentixafor PET-CT and Adrenal Vein Sampling in Patients with Typical Adenoma on CT Scan **eTable 5.** Concordance of Adrenal CT and Adrenal Vein Sampling **eTable 6.** Concordance of Gallium-68 Pentixafor PET-CT and Adrenal Vein Sampling Using Lateralization Index Based on Maximum Standardized Uptake Value at 40 min for Subtyping Diagnosis **eTable 7.** Clinical and Biochemical Characteristics of Patients With Unilateral Primary Aldosteronism Missed by Gallium-68 Pentixafor PET-CT **eTable 8.** Comparison of Patients With Unilateral Primary Aldosteronism Missed vs Not Missed by PET-CT **eTable 9.** Characteristics of Patient With Partial Biochemical Remission After Unilateral Adrenalectomy
eReferences.

Supplement 2.
Nonauthar Collaborators

Supplement 3.
Data Sharing Statement
