# Supplementary material for: Accuracy of Gallium-68 Pentixafor Positron Emission Tomography–Computed Tomography for Subtyping Diagnosis of Primary Aldosteronism
Source: JAMA Netw Open. 2023 Feb 16;6(2):e2255609. doi: 10.1001/jamanetworkopen.2022.55609 (PMC9936343; doi:10.1001/jamanetworkopen.2022.55609)
Supplement: Supplement 1. — eAppendix. Supplementary Methods eFigure 1. Scatter Plots Showing Correlation of Maximum Standardized Uptake Value With Adrenal Vein Sampling Parameters eFigure 2. Receiver Operating Characteristic Curve of Dominant Side of Maximum Standardized Uptake Value During Gallium-68 Pentixafor PET-CT for Diagnosis of Unilateral Primary Aldosteronism eFigure 3. Representative Images of Gallium-68 Pentixafor PET-CT Imaging in Patients With Primary Aldosteronism eTable 1. Proportion of Missing Data in Study Variables eTable 2. Diagnostic Accuracy of Adjusted Maximum Standardized Uptake Value at Different Cutoff Values eTable 3. Concordance of Gallium-68 Pentixafor PET-CT and Adrenal Vein Sampling in All Patients With PA eTable 4. Concordance of Gallium-68 Pentixafor PET-CT and Adrenal Vein Sampling in Patients with Typical Adenoma on CT Scan eTable 5. Concordance of Adrenal CT and Adrenal Vein Sampling eTable 6. Concordance of Gallium-68 Pentixafor PET-CT and Adrenal Vein Sampling Using Lateralization Index Based on Maximum Standardized Uptake Value at 40 min for Subtyping Diagnosis eTable 7. Clinical and Biochemical Characteristics of Patients With Unilateral Primary Aldosteronism Missed by Gallium-68 Pentixafor PET-CT eTable 8. Comparison of Patients With Unilateral Primary Aldosteronism Missed vs Not Missed by PET-CT eTable 9. Characteristics of Patient With Partial Biochemical Remission After Unilateral Adrenalectomy eReferences. [file jamanetwopen-e2255609-s001.pdf]

Supplemental Online Content

Hu J, Xu T, Shen H, et al; Chongqing Primary Aldosteronism Study (CONPASS) Group. Accuracy of gallium-68 pentixafor positron emission tomography–computed tomography for subtyping diagnosis of primary aldosteronism. *JAMA Netw Open*. 2023;6(2):e2255609. doi:10.1001/jamanetworkopen.2022.55609

eAppendix. Supplementary Methods

eFigure 1. Scatter Plots Showing Correlation of Maximum Standardized Uptake Value With Adrenal Vein Sampling Parameters

eFigure 2. Receiver Operating Characteristic Curve of Dominant Side of Maximum Standardized Uptake Value During Gallium-68 Pentixafor PET-CT for Diagnosis of Unilateral Primary Aldosteronism

eFigure 3. Representative Images of Gallium-68 Pentixafor PET-CT Imaging in Patients With Primary Aldosteronism

eTable 1. Proportion of Missing Data in Study Variables

eTable 2. Diagnostic Accuracy of Adjusted Maximum Standardized Uptake Value at Different Cutoff Values

eTable 3. Concordance of Gallium-68 Pentixafor PET-CT and Adrenal Vein Sampling in All Patients With PA

eTable 4. Concordance of Gallium-68 Pentixafor PET-CT and Adrenal Vein Sampling in Patients with Typical Adenoma on CT Scan

eTable 5. Concordance of Adrenal CT and Adrenal Vein Sampling

eTable 6. Concordance of Gallium-68 Pentixafor PET-CT and Adrenal Vein Sampling Using Lateralization Index Based on Maximum Standardized Uptake Value at 40 min for Subtyping Diagnosis

eTable 7. Clinical and Biochemical Characteristics of Patients With Unilateral Primary Aldosteronism Missed by Gallium-68 Pentixafor PET-CT

eTable 8. Comparison of Patients With Unilateral Primary Aldosteronism Missed vs Not Missed by PET-CT

eTable 9. Characteristics of Patient With Partial Biochemical Remission After Unilateral Adrenalectomy

eReferences.

This supplemental material has been provided by the authors to give readers additional information about their work.

eAppendix. Supplementary Methods

Study design and participants

The inclusion criteria were: aged 18 to 70 years, PA diagnosis confirmed by at least one confirmatory test, and consent to participate in the study. The exclusion criteria were: a) PA patients who met the guideline criteria for bypassing AVS [i.e. younger than 35 years, with typical APA characteristics (plasma aldosterone >30ng/dl, serum potassium <3.5 mEq/L, CT indicated unilateral 1cm low-density adenoma)[1]; b) failed adrenal vein cannulation during AVS; c) Subtyping diagnosis was inconclusive based on AVS results (e.g. aldosterone/cortisol ratio in bilateral adrenal veins lower than the peripheral vein, or missing data); d) pregnant or lactating women; e) patients with a history of uncontrolled malignant tumor; f) concurrent Cushing's syndrome [cortisol after 1mg dexamethasone suppression test >5μg/dl or cortisol after 1mg dexamethasone suppression test 1.8-5μg/dl plus adrenocorticotrophic hormone <10pg/ml[2]; g) diagnosis of familial hyperaldosteronism; h) imaging characteristics suggestive of pheochromocytoma or adrenal cortical carcinoma; i) unsuitable for surgery, such as heart failure with New York Heart Association (NYHA) class III or IV, severe anemia (Hemoglobin<6g/dL), stroke or acute coronary syndrome within 3 months, severe ascites and cirrhosis, estimated glomerulus filtration rate<30ml/min/m^2; j) alcohol or drug abuse and mental disorders.

Diagnosis of PA

All patients underwent PA screening by plasma aldosterone/renin ratio (ARR). The screening test was considered positive when the ARR was ≥ 3.16 $ng \cdot dl^{-1}/pg \cdot ml^{-1}$ [3,4]. Before screening, diuretic therapy, including mineralocorticoid receptor antagonists, was withdrawn for at least 4 weeks, and angiotensin-converting enzyme inhibitors, angiotensin-II receptor blockers and β-blockers were stopped for at least two weeks. Non-dihydropyridine calcium channel blockers and/or α-adrenergic blockers were allowed for uncontrolled hypertension. Samples for plasma renin concentration (PRC) and plasma aldosterone concentration (PAC) were collected in the morning after participants had been out of bed for at least 2 hours and after they had been seated for 15 minutes.

Patients who tested positive proceeded to confirmatory testing with a captopril challenge test (CCT) and seated saline infusion test (SSIT). PA was confirmed if at least one of the following criteria was met: a) PAC ≥11 ng/dl two hours after administration of 50 mg captopril[5]; b) PAC ≥8 ng/dl after the infusion of 2L normal saline[6]; c) if CCT or SSIT were in grey zone, i.e, PAC 8-11 ng/dl two hours after administration of 50 mg captopril or PAC 6-8 ng/dl after the infusion of 2L normal saline, fludrocortisone suppression test was performed and considered diagnostic of PA if PAC on the fourth day of fludrocortisone administration exceeded 6 ng/dl[1].

^{68}Ga-Pentixafor PET/CT scanning and image analysis

The pentixafor was purchased from Invivo Chemical Technology Co., Ltd (Guangzhou, China). A ^{68}Ge/^{68}Ga generator (China Isotope and Radiation Corporation, Beijing, China) was washed with 0.05 mmol/l hydrochloric acid, and ^{68}Ga-solution was obtained for radioactive labelling. Radiolabeling of ^{68}Ga-pentixafor was performed by adding 1 ml 0.25 mmol/l sodium acetate and 4 ml ^{68}Ga-solution (approximate 740 MBq) to a reactor with Pentixafor solution. The final pH was 3.5-4.5. The reaction mixture was heated to 80 °C for 10-15 min and the product was isolated by solid-phase extraction using 0.5 ml ethanol as eluent. The final product was diluted with saline and sterilized by passing through a 0.22-μm Millipore filter. The final product was sterile and pyrogen-free. The radiochemical purity of the ready-to-inject formulation as confirmed by radio–high-performance liquid chromatography and it

© 2023 Hu J et al. *JAMA Network Open.*

was >98%.

The patients had a normal diet with no special preparation before ^{68}Ga-Pentixafor PET/CT imaging. The dosage of intravenously injected ^{68}Ga-Pentixafor was calculated based on the patient's weight (1.85 MBq [0.05mCi]/kg). Local PET/CT scanning of the upper abdomen was performed on a hybrid PET/CT scanner (MI780, United Imaging Healthcare, Shanghai, China) at 10 minutes and 40 minutes after the injection of the intravenous tracer, respectively. Non-contrast CT (120 kV, 120 mA, images reconstructed with a slice thickness of 3 mm) were acquired over the upper abdomen and the shape and density of each adrenal gland was analyzed. Adrenal lesion included nodule (defined as round or oval, with smooth margins, well defined, ≥4mm in diameter) and hyperplasia (if adrenal gland thickness measured ≥10 mm in diameter). A PET scan in 3D acquisition mode was immediately performed after the CT scan, and PET images of the adrenal region were acquired over 5 minutes using one bed position. All the obtained data were transferred to the post-processing workstation (Version R002, United Imaging Healthcare, Shanghai, China). CT data was used for PET attenuation correction and PET data were reconstructed using the ordered subset expectation maximization algorithm (two iterations and 20 subsets). After the reconstruction was completed, the post-processing and fusion software of United Imaging was used for image analysis.

The PET/CT image interpretation was based on visual and semi-directional analysis. The shape and density of adrenal glands were analyzed. Lesions showed on CT or those with no abnormality on CT but suspected increased tracer uptake on PET were located as regions of interest, and maximal standardized uptake value (SUVmax) was measured in these regions. For adrenal glands with neither morphological changes nor increased tracer uptake, SUVmax within each adrenal gland was also recorded. Average SUVmax of five round spheres with a diameter of 2 cm was selected from the liver as the whole-body background.

Lateralization index (LI) based on SUVmax at 10min and 40min, dominant side of SUVmax at 10min and 40min, and dominant side of SUVmax adjusted by liver (DSAL) at 10min and 40min were calculated for the diagnostic accuracy analysis. The side with higher SUVmax in both adrenal glands is the dominant side. LI based on SUVmax was defined as (SUVmax of dominant side)/(SUVmax of nondominant side). SUVmax adjusted by liver was defined as (SUVmax of adrenal)/(SUVmax of liver). Dominant side of SUVmax adjusted by liver (DSAL) was defined as (SUVmax of dominant side in adrenal)/(SUVmax of liver).

The PET/CT images were evaluated by two experienced nuclear medical doctors who were blinded to the AVS result. A consensus report was generated commenting on the location of the lesion and the value of SUVmax.

Adrenal vein sampling (AVS)

With local anesthesia, a sheath was inserted into the right femoral vein followed by catheter insertion in the adrenal veins. Blood samples in right and then left adrenal veins were collected sequentially from right and left adrenal veins. Three tubes of blood in each adrenal vein were collected consecutively, and one tube of blood in the inferior vena cava was collected immediately after the collection of each side of the adrenal vein blood. The average results of three adrenal vein blood samples were used for index calculation.

The selectivity index was defined as cortisol (adrenal vein) / cortisol (peripheral vein) and successful cannulation of the adrenal veins were defined as selectivity index ≥ 2. The LI was defined as [aldosterone-cortisol ratio of dominant adrenal vein)]/[aldosterone-cortisol ratio of nondominant adrenal vein] while contralateral suppression was defined as [aldosterone-cortisol ratio of

© 2023 Hu J et al. *JAMA Network Open.*

non-dominant adrenal vein] < [aldosterone-cortisol ratio of peripheral vein]. A diagnosis of UPA was made if LI ≥ 4 or LI 2-4 in combination with contralateral suppression or CT showing a typical adenoma on the dominant side, while those with LI < 2 or LI 2-4 without meeting the above criteria were diagnosed as BPA[7,8].

Measurements and assay methods

Blood pressure was measured according to the European Society of Hypertension/European Society of Cardiology Guidelines for the management of arterial hypertension. Patients were seated in a quiet room for blood pressure measurement, which was completed with an electronic sphygmomanometer. Use a standard bladder cuff for most patients, but the cuff could be changed to a larger or smaller one according to the size of the patient's arm. Blood pressure was measured in both arms initially, and the arm with the higher reading was used for subsequent measurements of blood pressure. The device was set to start the first measurement after 5 minutes of rest. At least 2 measurements were taken 2 minutes apart. Additional measurements were performed if the first two measurements differed significantly and the average blood pressure was calculated. The sphygmomanometers were regularly recalibrated according to manufacturers' instructions.

PRC and PAC were measured with an automated chemiluminescence immunoassay (LIAISON; DiaSorin, Italy). For aldosterone, the within-run coefficients of variation were 3.5% at 6.8 ng/dl and 1.8% at 28.8 ng/dl. The total coefficients of variation were 9.5% at 6.8 ng/dl and 5.6% at 28.8 ng/dl. The analytical sensitivity (defined as the minimum detectable concentration that could be distinguished from zero) for the PAC was 2.2 ng/dl. For PRC, the within-run coefficients of variation were 12.4% at 8.4pg/ml and 4.7% at 164.7pg/ml. The total coefficients of variation were 0.6% at 8.4pg/ml and 1.7% at 164.7pg/ml. The analytical sensitivity for the PRC was 0.3pg/ml (the lowest detectable level). Quality control was performed every day in the laboratory.

Statistical analysis

Our study aimed to assess the diagnostic accuracy of ^{68}Ga-Pentixafor PET/CT for subtyping diagnosis of primary aldosteronism. Based on the previous report[9], the receiver operating characteristic (ROC) curve could be used to assess the accuracy of a diagnostic test. Therefore, the area under ROC (AUC) was selected as the measure of main importance in our study. Power Analysis and Sample Size software 11 (PASS 11) was used to calculate the sample size. A sample of 40 from the positive group (i.e. UPA group) and 60 from the negative group (i.e. BPA group) achieves 84% power to detect a difference of 0.15 between the area under the receiver–operator characteristics curve (AUC) under the null hypothesis of 0.70 and an AUC under the alternative hypothesis of 0.85 using a two-sided z-test at a significance level of 0.05.

AUC of 0.7 represents the AUC0' (AUC|H0), which is the adjusted AUC under the null hypothesis. We assumed that if the diagnostic accuracy of PET/CT is similar to the conventional CT, it will meet the criterion of null hypothesis. In a previous published systematic review[10], the AUC of conventional CT to differentiate unilateral primary aldosteronism (UPA) from bilateral primary aldosteronism (BPA) is 0.67, thus we estimated the AUC0' as 0.7. AUC of 0.85 represents the AUC1' (AUC|H1), which is the adjusted AUC the alternative hypothesis. Based on a previous report[11], using SUVmax to differentiate UPA from nonfunctional adrenal adenoma, the AUC was 0.88. Considering that it might be easier to distinguish UPA from nonfunctional tumors than UPA from BPA, we assumed the AUC1' as 0.85.

AUC, sensitivity, specificity, positive predictive value, negative predictive value, and Youden index were calculated to evaluate the accuracy. The AUC was calculated by MedCalc software 19.5.2. Other

© 2023 Hu J et al. *JAMA Network Open.*

statistical analyses were performed with SPSS Statistics Version 23.0 (IBM, Armonk, New York). The distribution of the data was analyzed with the Kolmogorov-Smirnov test. Normally distributed variables were expressed as the mean and standard deviation (SD) and analyzed by Student's t test; variables with a skewed distribution were expressed as median (interquartile range) and analyzed by the Mann-Whitney U test; categorical variables were described as percentages analyzed by the $\chi 2$ test or Fisher's Exact Test.

The sensitivity and specificity were calculated as follows: sensitivity=true positive/(true positive + false negative); true positive=the number of UPA patients (diagnosed by AVS) higher than the cutoff of PET/CT; false negative=the number of UPA patients (diagnosed by AVS) lower than the cutoff of PET/CT; specificity= true negative/(true negative+ false positive), true negative= the number of BPA patients (diagnosed by AVS) lower than the cutoff of PET/CT; false positive= the number of BPA patients (diagnosed by AVS) higher than the cutoff of PET/CT; false positive(FP)= the number of BPA patients (diagnosed by AVS) higher than the cutoff of PET/CT. PPV(positive predictive value) =(TP/FP+TP)×100%; NPV(negative predictive value)=(TN/FN+TN)×100%;YI(youden index)= sensitivity+ specificity-1

Missing data were imputed with the multivariate imputation by chained equations algorithm (https://amices.org/mice/). Regression models were utilized to predict the missing values. The analyses were conducted using R version 4.0.1 (R Project for Statistical Computing, Vienna, Austria).

© 2023 Hu J et al. *JAMA Network Open.*

eFigure 1. Scatter Plots Showing Correlation of Maximum Standardized Uptake Value With Adrenal Vein Sampling Parameters

A

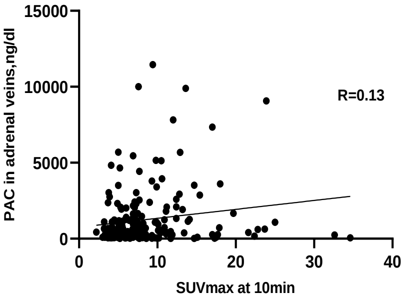

B

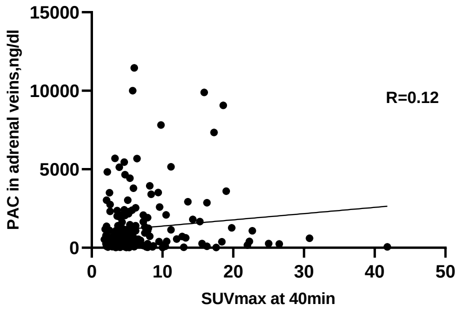

C

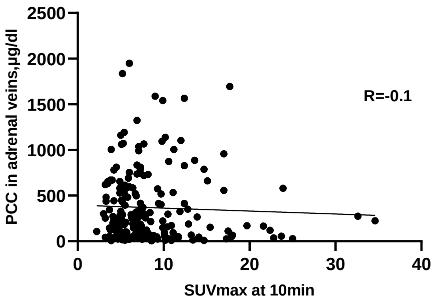

D

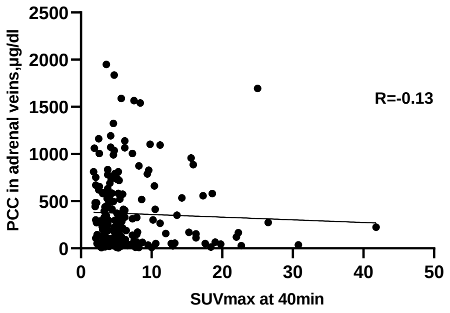

E

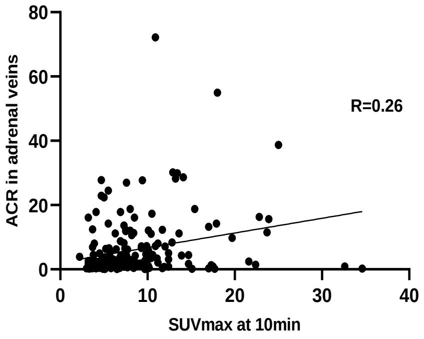

F

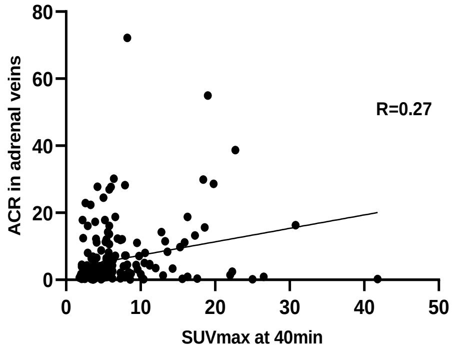

G

H

© 2023 Hu J et al. *JAMA Network Open.*

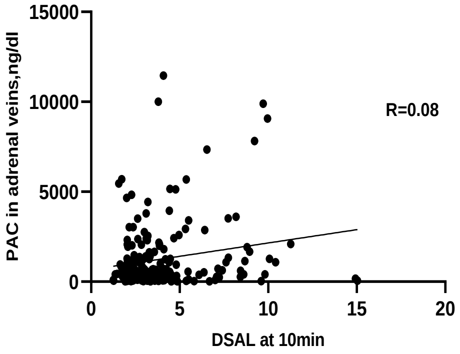

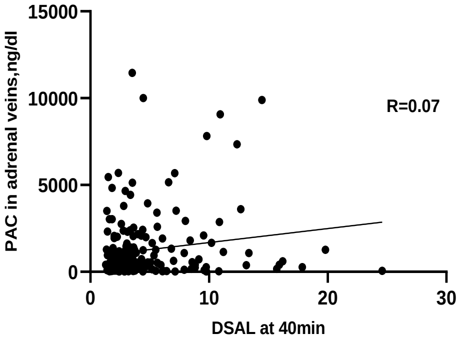

I

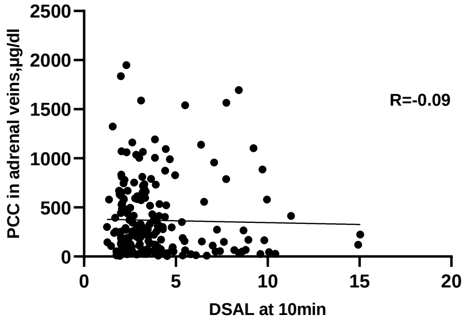

J

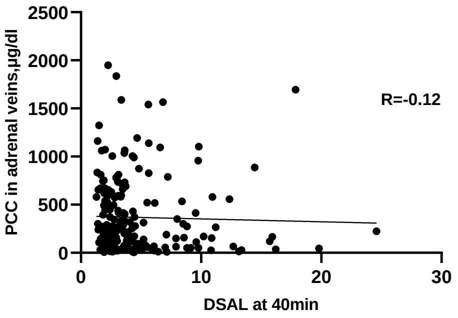

K

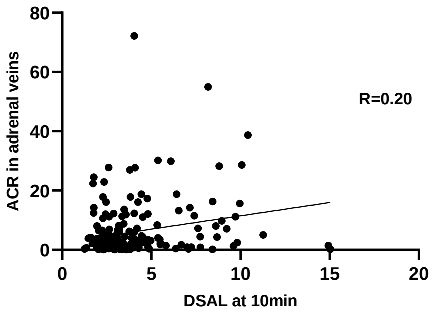

L

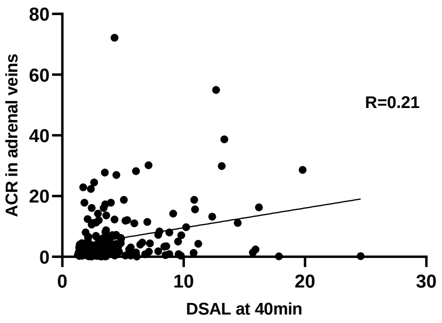

M

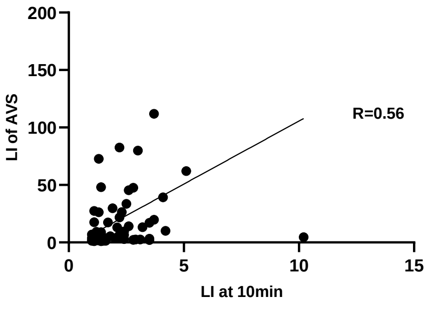

N

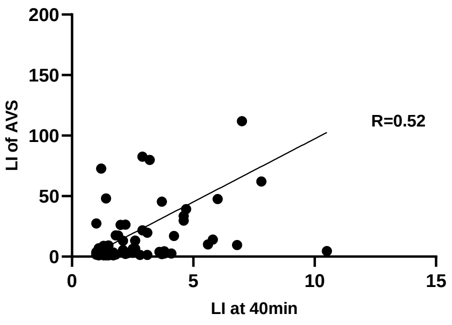

© 2023 Hu J et al. *JAMA Network Open.*

eFigure 2. Receiver Operating Characteristic Curve of Dominant Side of Maximum Standardized Uptake Value During Gallium-68 Pentixafor PET-CT for Diagnosis of Unilateral Primary Aldosteronism

A

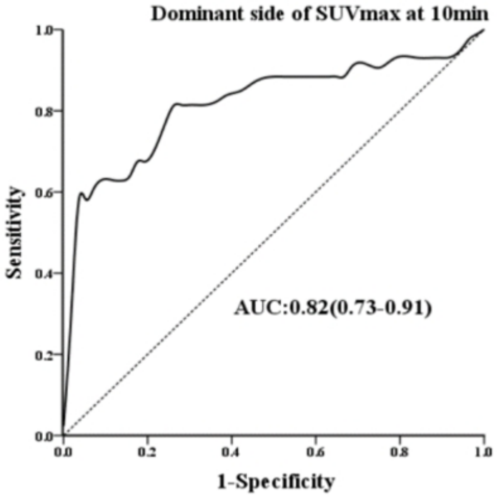

B

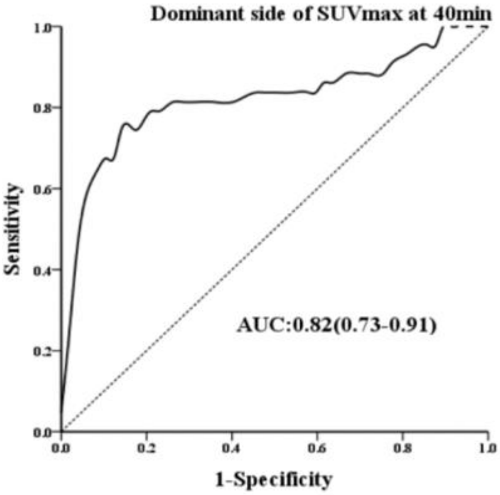

ROC: receiver–operator characteristic curves; UPA: unilateral primary aldosteronism; AUC: area under ROC curves.

© 2023 Hu J et al. *JAMA Network Open.*

eFigure 3. Representative Images of Gallium-68 Pentixafor PET-CT Imaging in Patients With Primary Aldosteronism

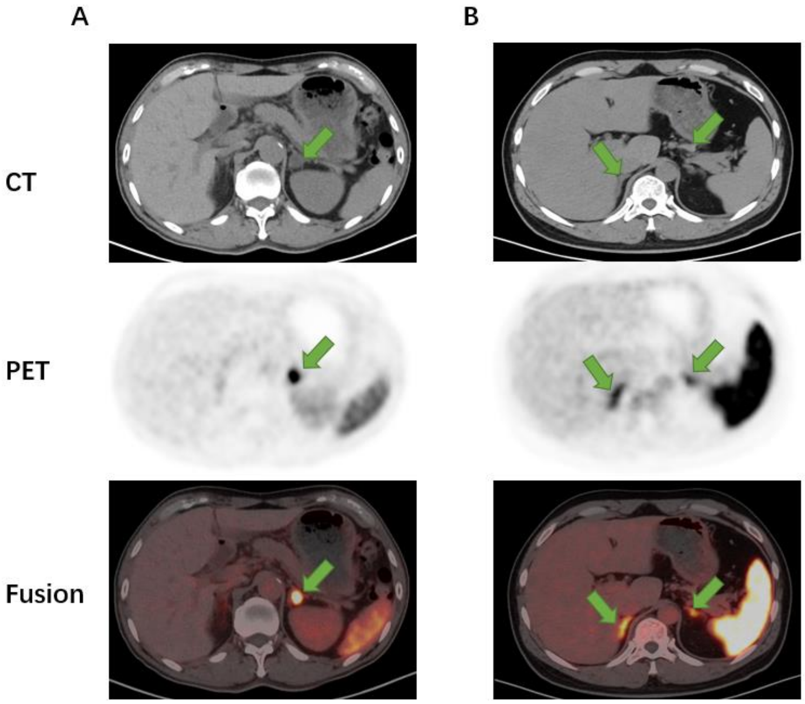

(A) ^{68}Ga-pentixafor imaging in a 62-year-old male patient with unilateral primary aldosteronism 10min after injection of ^{68}Ga-pentixafor; he had a history of hypertension for eight years, with serum potassium 1.8 mEq/L, plasma aldosterone concentration 15.2 ng/dl, plasma renin concentration 0.3pg/ml, aldosterone/renin ratio 48.0 ng·dl^{-1}/pg·ml^{-1}, plasma aldosterone concentration post captopril challenge test 31.2ng/dl; PET/CT showed a 1.6 × 1.2 cm nodule with intense uptake (SUVmax 13.6) in the left adrenal gland, and the right adrenal showed no morphological abnormality or increased uptake (SUVmax 5.1), and the lateralization index based on SUVmax was 4.1; AVS revealed the over-secretion of aldosterone in the left adrenal (with lateralization index of 39.2); his blood pressure and serum potassium returned to normal range after surgery. (B) ^{68}Ga-pentixafor imaging in a 46-year-old male patient with bilateral primary aldosteronism 10min after injection of ^{68}Ga-pentixafor; he had a history of hypertension for eight years, with serum potassium 2.7 mEq/L, plasma aldosterone concentration 10.5ng/dl, plasma renin concentration 2.8pg/ml, aldosterone/renin ratio 3.7 ng·dl^{-1}/pg·ml^{-1}, plasma aldosterone post fludrocortisone suppression test 10.5 ng/dl; PET/CT showed bilateral adrenal hyperplasia with slightly increased uptake (SUVmax 9.7 in right adrenal, SUVmax 9.8 in right adrenal), and the lateralization index based on SUVmax was 1.01; AVS revealed no lateralization in the two adrenals (with lateralization index of 1.7). Arrows indicate the lesion.

© 2023 Hu J et al. *JAMA Network Open.*

eTable 1. Proportion of Missing Data in Study Variables

	UPA(n=43)	BPA(n=57)
Age, %	0	0
Sex, %	0	0
BMI, %	0	0
SBP, %	0	0
DBP, %	0	0
Serum K^+, %	0	0
PAC, %	0	0
PRC, %	0	0
PAC post-CCT, %	5	0
PAC post-SSIT, %	9	4
Adernal CT scan, %	0	0
Dominant side of SUVmax at 10min, %	0	0
Dominant side of 10min SUVmax adjusted by liver, %	0	0
Dominant side of SUVmax at 40min, %	0	0
Dominant side of 40min SUVmax adjusted by liver, %	0	0
Non-dominant side of SUVmax at 10min, %	0	0
Non-dominant side of 10min SUVmax adjusted by liver, %	0	0
Non-dominant side of SUVmax at 40min, %	0	0
Non-dominant side of 40min SUVmax adjusted by liver, %	0	0
LI based on SUVmax at 10min, %	0	0
LI based on SUVmax at 40min, %	0	0
Dominant side of PAC, %	0	0
Dominant side of PCC, %	0	0
Dominant side of ACR, %	0	0
Non-dominant side of PAC, %	0	0
Non-dominant side of PCC, %	0	0
Non-dominant side of ACR, %	0	0
LI based on AVS, %	0	0

UPA: unilateral primary aldosteronism; BPA: bilateral primary aldosteronism; CCT:captopril challenge test;SSIT: Seated saline infusion test; SUVmax: maximum standardized uptake value; PAC: plasma aldosterone concentration; PCC: plasma cortisol concentration; LI: lateralization index; ACR: aldosterone-to-cortisol ratio, AVS: adrenal vein samplin

© 2023 Hu J et al. *JAMA Network Open.*

eTable 2. Diagnostic Accuracy of Adjusted Maximum Standardized Uptake Value at Different Cutoff Values

	Cutoff	TP	FP	FN	TN	Sensitivity(95%CI)	Specificity(95%CI)	YI	PPV(95%CI)	NPV(95%CI)
DSAL at 10min	4.7	33	6	10	51	0.77(0.61-0.88)	0.89(0.78-0.96)	0.66	0.85(0.69-0.94)	0.84(0.72-0.92)
	5.4	28	3	15	54	0.65(0.49-0.79)	0.95(0.85-0.99)	0.60	0.90(0.74-0.98)	0.78(0.67-0.87)
	10.8	3	0	40	57	0.07(0.01-0.19)	1.00(0.94-1.00)	0.07	1.00(0.29-1.00)	0.59(0.48-0.69)
DSAL at 40min	5.1	35	8	8	49	0.81(0.67-0.92)	0.86(0.74-0.94)	0.67	0.81(0.67-0.92)	0.86(0.74-0.94)
	6.9	29	3	14	54	0.67(0.51-0.81)	0.95(0.85-0.99)	0.62	0.91(0.75-0.98)	0.79(0.68-0.88)
	13.9	7	0	36	57	0.16(0.07-0.31)	1.00(0.94-1.00)	0.16	1.00(0.59-1.00)	0.61(0.51-0.71)
Dominant side	5.7	40	44	3	13	0.93(0.81-0.99)	0.23(0.13-0.36)	0.13	0.48(0.37-0.59)	0.81(0.54-0.96)
of SUVmax at 10min	9.3	35	15	8	42	0.81(0.67-0.92)	0.74(0.60-0.84)	0.55	0.70(0.55-0.82)	0.84(0.71-0.93)
	12.6	23	1	20	56	0.53(0.38-0.69)	0.98(0.91-1.00)	0.51	0.96(0.79-1.00)	0.74(0.62-0.83)
Dominant side	3.8	40	46	3	11	0.93(0.81-0.99)	0.19(0.10-0.32)	0.12	0.47(0.36-0.58)	0.79(0.49-0.95)
of SUVmax at 40min	7.8	32	8	11	49	0.74(0.59-0.86)	0.86(0.72-0.94)	0.60	0.80(0.64-0.91)	0.82(0.70-0.90)
	14.8	16	1	27	56	0.37(0.23-0.53)	0.98(0.91-1.00)	0.35	0.94(0.71-1.00)	0.67(0.56-0.77)

DSAL: Dominant side of SUVmax adjusted by liver; SUVmax: maximum standardized uptake value; TP: True Positive, FP: False Positive, FN: False Negative, TN: True Negative, PPV: positive predictive value, NPV: negative predictive value, YI: youden index.

© 2023 Hu J et al. *JAMA Network Open.*

eTable 3. Concordance of Gallium-68 Pentixafor PET-CT and Adrenal Vein Sampling in All Patients With PA

Subtyping diagnosis based on AVS	Subtyping diagnosis based on ^{68}Ga-Pentixafor PET/CT			Concordance rate of AVS and PET/CT, % (n)
	Left	Right	Bilateral	
Left(n=24)	22	0	2	
Right(n=19)	0	11	8	
Bilateral(n=57)	0	0	57	
				90.0% (90/100)

PET/CT were using the LI based on SUVmax at 10min ≥1.65.

© 2023 Hu J et al. *JAMA Network Open.*

eTable 4. Concordance of Gallium-68 Pentixafor PET-CT and Adrenal Vein Sampling in Patients with Typical Adenoma on CT Scan

Subtyping diagnosis based on AVS	Subtyping diagnosis based on ^{68}Ga-Pentixafor PET/CT			Concordance rate of AVS and PET/CT, % (n)
	Left	Right	Bilateral	
Left(n=17)	17	0	0	
Right(n=8)	0	8	0	
Bilateral(n=15)	0	0	15	
				100%(40/40)

PET/CT were using the LI based on SUVmax at 10min ≥1.65. Typical adenoma was defined as unilateral adrenal nodules greater than 10 mm

© 2023 Hu J et al. *JAMA Network Open.*

eTable 5. Concordance of Adrenal CT and Adrenal Vein Sampling

Subtyping diagnosis based on AVS	Subtyping diagnosis based on CT			Concordance of AVS and CT, % (n)
	Left	Right	Bilateral*	
Left(n=24)	21	0	3	
Right(n=19)	2	14	3	
Bilateral(n=58)	34	3	20	
				55.0% (55/100)

*Bilateral normal or bilateral lesion

© 2023 Hu J et al. *JAMA Network Open.*

eTable 6. Concordance of Gallium-68 Pentixafor PET-CT and Adrenal Vein Sampling Using Lateralization Index Based on Maximum Standardized Uptake Value at 40 min for Subtyping Diagnosis

Subtyping diagnosis based on AVS	Subtyping diagnosis based on ^{68}Ga-Pentixafor PET/CT			Concordance of AVS and PET/CT, % (n)
	Left	Right	Bilateral	
Left(n=24)	23	0	1	
Right(n=19)	0	14	5	
Bilateral(n=57)	5	0	52	
				89.0% (89/100)

PET/CT were using the LI based on SUVmax at 10min ≥1.57.

© 2023 Hu J et al. *JAMA Network Open.*

eTable 7. Clinical and Biochemical Characteristics of Patients With Unilateral Primary Aldosteronism Missed by Gallium-68 Pentixafor PET-CT

Patient Number	Age (years)	Sex	Serum potassium (mEq/L) *†	PAC (ng/dl) ‡	PRC (pg/ml) §	ARR (ng·dl⁻¹/pg·ml⁻¹) ‡§	PAC post CCT (ng/dl) ‡	PAC post SIT (ng/dl) ‡	CT findings side	CT findings lesion (mm)	Post-DST cortisol (μg/dl) ‖	AVS SI Left	AVS SI Right	AVS LI	AVS CLR	LI based on SUVmax at 10min
No.045	30s	F	4.1	17.60	0.73	24.18	20.30	15.80	left	hyperplasia	0.50	70.33	7.52	9.21	1.54	1.23
No.047	40s	F	3.3	11.80	1.63	7.23	7.30	8.96	right	nodule(8mm)	1.20	2.35	5.98	2.90	2.45	1.39
No.052	50s	F	3.1	14.00	0.35	39.50	14.50	4.86	left	nodule(8mm)	0.69	13.45	16.39	6.85	0.76	1.04
No.053	50s	F	3.3	16.10	1.34	12.01	10.90	16.20	bilateral	nodule(18mm)	0.87	2.88	2.74	17.64	0.65	1.10
No.057	60s	M	3.4	20.40	1.45	14.08	31.70	10.70	bilateral	right hyperplasia and left nodule(10mm)	0.77	23.21	2.35	72.83	0.10	1.32
No.074	20s	M	4.2	13.40	1.60	8.37	13.30	9.34	right	nodule(8mm)	0.45	33.03	25.43	26.22	0.08	1.33
No.102	30s	M	3.6	15.40	0.33	46.79	9.98	8.54	bilateral	left hyperplasia and right nodule(11mm)	0.95	8.15	46.47	3.39	0.95	1.41
No.103	60s	M	3.6	15.20	3.22	4.73	16.40	10.30	normal	normal	0.78	20.95	14.10	5.32	0.63	1.19

© 2023 Hu J et al. *JAMA Network Open.*

No.112	30s	F	3.9	15.03	1.03	14.57	29.20	10.50	right	nodule(5mm)	1.71	16.04	25.38	27.36	0.25	1.11
No.119	60s	M	2.5	33.2	1.22	27.32	25.10	15.50	bilateral	left hyperplasia and right nodule(9mm)	1.16	22.38	10.72	48.16	0.19	1.35

F: female; M:male; LI: lateralization index;PAC: plasma aldosterone concentration;PRC:plasma reninconcentration; ARR:Ratio of PAC to PRC; CCT:captopril challenge test;SIT: saline infusion test; DST: dexamethasone suppression test ; SI:selectivity index; CLR:contralateral ratio; * Serum potassium was the lowest level in the medical history before treatment of hypokalemia.

†: To convert mEq/L to mmol/L, multiply by 1.

‡: To convert ng/dl to pmol/L, multiply by 27.74.

§:To convert pg/ml to pmol/L, multiply by 0.0237.

||: To convert μg/dl to nmol/l, multiply by 27.588.

© 2023 Hu J et al. *JAMA Network Open.*

eTable 8. Comparison of Patients With Unilateral Primary Aldosteronism Missed vs Not Missed by PET-CT

Characteristic	Missed(n=10)	Correct(n=33)	P Value
Age(y)	47(34-62)	47(38-54)	0.99
Female, n (%)	5(50)	15(45)	0.80
Serum K^+ (mEq/L)*†	3.5(3.3-4.0)	3.3(2.6-3.6)	0.17
PAC (ng/dl)‡	15.3(13.9-18.3)	28.2(21.5-38.9)	0.001
PRC (pg/ml)§	1.3(0.6-1.6)	1.0(0.4-4.2)	0.97
ARR ($ng·dl^{-1}/pg· ml^{-1}$)‡§	14.3(8.1-30.4)	30.8(8.1-77.6)	0.34
PAC post-CCT (ng/dl)‡	15.5(10.7-26.1)	29.1(19.1-38.1)	0.003
PAC post-SSIT (ng/dl)‡	10.4(8.9-15.6)	22.8(15.1-33.7)	<0.001
Post-DST cortisol(μg/dl)||	0.8(0.6-1.1)	1.0(0.7-1.4)	0.15
Diameter of adrenal nodule on CT(mm)	8.5(8-10.8)	14(11-17)	0.01
Left SI of AVS	18.5(6.8-25.7)	17.2(7.2-31.1)	0.94
Right SI of AVS	12.4(5.2-25.4)	26.8(14.9-35.3)	0.03
LI of AVS	13.4(4.8-32.6)	13.6 (4.5-40.8)	0.87
CLR of AVS	0.6(0.2-1.1)	0.3(0.1-0.6)	0.26

LI: lateralization index;PAC: plasma aldosterone concentration;PRC:plasma reninconcentration; ARR:Ratio of PAC to PRC; CCT:captopril challenge test;SIT: saline infusion test; DST: dexamethasone suppression test; SI:selectivity index; CLR:contralateral ratio; * Serum potassium was the lowest level in the medical history before treatment of hypokalemia.†: To convert mEq/L to mmol/L, multiply by 1.

‡: To convert ng/dl to pmol/L, multiply by 27.74.

§:To convert pg/ml to pmol/L, multiply by 0.0237.

||: To convert μg/dl to nmol/l, multiply by 27.588.

© 2023 Hu J et al. *JAMA Network Open.*

eTable 9. Characteristics of Patient With Partial Biochemical Remission After Unilateral Adrenalectomy

Patient	Age		Sex	Serum		PAC	PRC	ARR		PAC
										post
Number				potassium						CCT
	(years)			(mEq/L) *		(ng/dl) †	(pg/ml) ‡	(ng·dl^{-1}/pg·ml^{-1}) †‡		(ng/dl) †
No.006	30s		M	2.6		27.30	0.88	31.03		19.40
	PAC		CT				AVS			
	post		findings			SI		LI		CLR
	SIT		side	lesion		Left	Right			
	(ng/dl) †			(mm)						
	13.70		left	Multiple nodules		20.19	44.00	6.46		0.23
	Post-			Follow-up after adrenalectomy						
	DST		Serum	Serum		PAC	PRC	ARR		PAC
	cortisol									post
			potassium	creatinine						CCT
	(μg/dl) §		(mEq/L) *	(mg/dl)||		(ng/dl) †	(pg/ml) ‡	(ng·dl^{-1}/pg·ml^{-1}) †‡		(ng/dl) †
	0.68		4	0.84		23.30	2.99	7.79		16.40

M:male; LI: lateralization index;PAC: plasma aldosterone concentration;PRC:plasma reninconcentration; ARR:Ratio of PAC to PRC; CCT:captopril challenge test;SIT: saline infusion test; DST: dexamethasone suppression test; SI:selectivity index; CLR:contralateral ratio;

*:To convert mEq/L to mmol/L, multiply by 1

†:To convert ng/dl to pmol/L, multiply by 27.74.

‡:To convert pg/ml to pmol/L, multiply by 0.0237.

§:To convert µg/dl to nmol/l, multiply by 27.588.

||: To convert mg/dl to µmol/l, multiply by 88.4.

© 2023 Hu J et al. *JAMA Network Open.*

eReferences

1. Funder JW, Carey RM, Mantero F, et al. The Management of Primary Aldosteronism: Case Detection, Diagnosis, and Treatment: An Endocrine Society Clinical Practice Guideline. *J Clin Endocrinol Metab.* 2016;101(5):1889-1916.
2. Yanase T, Oki Y, Katabami T, et al. New diagnostic criteria of adrenal subclinical Cushing's syndrome: opinion from the Japan Endocrine Society. *Endocr J.* 2018;65(4):383-393.
3. Ma L, Song Y, Mei M, et al. Age-Related Cutoffs of Plasma Aldosterone/Renin Concentration for Primary Aldosteronism Screening. *Int J Endocrinol.* 2018;2018:8647026.
4. Wang K, Hu J, Yang J, et al. Development and Validation of Criteria for Sparing Confirmatory Tests in Diagnosing Primary Aldosteronism. *J Clin Endocrinol Metab.* 2020;105(7).
5. Song Y, Yang S, He W, et al. Confirmatory Tests for the Diagnosis of Primary Aldosteronism. *Hypertension.* 2018;71(1):118-124.
6. Thuzar M, Young K, Ahmed AH, et al. Diagnosis of Primary Aldosteronism by Seated Saline Suppression Test-Variability Between Immunoassay and HPLC-MS/MS. *J Clin Endocrinol Metab.* 2020;105(3).
7. Rossi GP, Auchus RJ, Brown M, et al. An expert consensus statement on use of adrenal vein sampling for the subtyping of primary aldosteronism. *Hypertension.* 2014;63(1):151-160.
8. Monticone S, Viola A, Rossato D, et al. Adrenal vein sampling in primary aldosteronism: towards a standardised protocol. *The Lancet Diabetes & Endocrinology.* 2015;3(4):296-303.
9. Hanley JA, McNeil BJ. A method of comparing the areas under receiver operating characteristic curves derived from the same cases. *Radiology.* 1983;148(3):839-843.
10. Kempers MJ, Lenders JW, van Outheusden L, et al. Systematic review: diagnostic procedures to differentiate unilateral from bilateral adrenal abnormality in primary aldosteronism. *Ann Intern Med.* 2009;151(5):329-337.
11. Ding J, Zhang Y, Wen J, et al. Imaging CXCR4 expression in patients with suspected primary hyperaldosteronism. *Eur J Nucl Med Mol Imaging.* 2020;47(11):2656-2665.

© 2023 Hu J et al. *JAMA Network Open.*
