# Supplementary material for: Accuracy of Gallium-68 Pentixafor Positron Emission Tomography–Computed Tomography for Subtyping Diagnosis of Primary Aldosteronism
Source: JAMA Netw Open. 2023 Feb 16;6(2):e2255609. doi: 10.1001/jamanetworkopen.2022.55609 (PMC9936343; doi:10.1001/jamanetworkopen.2022.55609)
Supplement: Supplement 3. — Data Sharing Statement [file jamanetwopen-e2255609-s003.pdf]

Data Sharing Statement

Hu. Accuracy of Gallium-68 Pentixafor Positron Emission Tomography-Computed Tomography for Subtyping Diagnosis of Primary Aldosteronism. *JAMA Netw Open*. Published February 16, 2023. doi:10.1001/jamanetworkopen.2022.55609

Data
Data available: No
